# Fundamentals of SARS-CoV-2 Biosensors

**DOI:** 10.3390/bios12100880

**Published:** 2022-10-17

**Authors:** Carlos Torres-Torres, Blanca Estela García-Pérez

**Affiliations:** 1 Sección de Estudios de Posgrado e Investigación, Escuela Superior de Ingeniería Mecánica y Eléctrica Unidad Zacatenco, Instituto Politécnico Nacional, Mexico City 07738, Mexico; 2 Departamento de Microbiología, Escuela Nacional de Ciencias Biológicas, Instituto Politécnico Nacional, Mexico City 11340, Mexico

A beautiful topic in its essence and content is represented by the powerful assistance of sensing methods and techniques for automatically revealing biological agents and biological functions in this era. This Special Issue entitled “Fundamentals of SARS-CoV-2 Biosensors” has been mainly devoted to simultaneously integrate essential and cutting-edge discoveries in the role of coronavirus detection and identification of molecules of immune response as the antibodies neutralizing. This collection of papers is presented as a step towards the development of new alternatives related to current challenges in biosensors emerging for one of the major pandemics of this millennium up to date.

Wei Yin Lim, Boon Leong Lan and Narayanan Ramakrishnan have presented a review on three particular types of biosensors employed for detecting SARS-CoV-2 based on surface plasmon resonance effects, electrochemical measurements and field-effect transistors. Sensing principles and confrontation of the advantages and limitations of these sensors were analyzed. The use of biorecognition elements and plasmonic nanomaterials are proposed in order to improve the sensitivity of the biosensors [1].

Kseniya V. Serebrennikova et al. presented a lateral flow immunoassay (LFIA) including the plasmonic advantages of gold nanoparticles and nanostructures with immobilized antibodies and 4-mercaptobenzoic acid as surface-enhanced Raman scattering (SERS) nanotag. A systematic study of the compositions of SERS nanotags was carried out promoting the acceleration of the typical response time exhibited by LFIA by the incorporation of SERS effects in membrane immuno-analytical systems [2].

Tao Peng et al. showed an investigation about LFIA conducted by both colloidal gold nanoparticles and copper deposition-induced signal amplification. The LFIA coupled with the copper and gold nanostructures yield the enhancement of efficiency and sensitivity in the biosensing processes [3].

Zhanwei Liang et al. developed a point-of-care bicolor LFIA for detecting neutralizing (Nab) antibody against SARS-CoV-2 without sample pretreatment. It is indicated the importance of the principle of NAb-mediated blockage on the interaction between the receptor binding domain of the spike protein and the angiotensin-converting enzyme 2. Red and blue latex microspheres were employed to carry out the measurement and control lines in order to reduce the error bar usually given by monitoring and interpretation of single-colored line data [4]. 

Elda A. Flores-Contreras et al. reported an analysis about comparative microfluidic platforms employed for SARS-CoV-2 testing. The systems studied were classified according to three different molecules to be detected, which were nucleic acid, antigens, and anti-SARS-CoV-2 antibodies. This manuscript includes a critical report about commercially available alternatives based on microfluidic processes [5].

Wilson A. Ameku et al. designed an electrochemical biosensor for detecting serological immunoglobulin G (IgG) antibodies in sera against spike proteins. How the capture of SARS-CoV-2-specific IgGs generates the formation of an immunocomplex is highlighted. Rapid trace signatures from square-wave voltammetry and the generation of hydroquinone are claimed. Absence of cross-reaction between SARS-CoV-2 and Chagas disease, Chikungunya, Leishmaniosis, Dengue, or new variants of SARS-CoV-2 is guaranteed by this method [6].

Sabine Szunerits, Hiba Saada, Quentin Pagneux and Rabah Boukherroub underlined the powerful potential of portable surface plasmon resonance systems for viral diagnostic and monitoring. A clear review focused on modern biosensing based on surface plasmon resonance phenomena to convert the receptor-binding event of SARS-CoV-2 viral particles into measurable signals was developed. Advantages and details about the key roles of plasmonics for virus particle detection as well as viral protein sensing were analyzed [7].

Geert Besselink et al. proposed a photonic integrated circuit based on an asymmetric Mach–Zehnder interferometer for SARS-CoV-2 biosensing. Attractive advantages of photonics featuring tunable and high analytical sensitivity, signal multiplexing technology, small size, portability, and the potential for high-volume manufacturing were described. Spike proteins, receptor-binding domain, and nucleocapsid proteins as target antigens were experimentally tested in this work [8]. 

Chia-Hsuan Cheng et al. mentioned the crucial role to detect the amount of anti-SARS-CoV-2 S protein antibodies prior to vaccination. This is regarding the fact that vaccines, apart from inducing antibody production, could cause and adverse effects such as myocarditis, blood clots, muscle pain and fatigue, among others. A shear-horizontal surface acoustic wave biosensor coated with SARS-CoV-2 spike protein is presented in this research to quantify the amount of anti-SARS-CoV-2 S protein antibodies from finger blood. It is reported that mRNA vaccines, such as Moderna or BNT, can originate stronger effects derived by higher concentrations of total anti-SARS-CoV-2 S protein antibodies compared with adenovirus vaccines [9].

Kritika Srinivasan Rajsri et al. described their findings on a rapid quantitative point-of-care serological assay for quantifying anti-SARS-CoV-2 antibodies. the potential use of this proposed system for the timing of booster shots and measuring the level of cross-reactive antibodies is pointed out. A lab-on-a-chip ecosystem is presented as a strategy to easily quantitate the humoral protection against COVID-19. The design proposed includes characteristics for the diagnostic timeline of the disease, seroconversion, and vaccination response spanning multiple doses of immunization in just one test [10]. 

Nayeli Shantal Castrejón-Jiménez et al. summarized the progress and dynamic evolution of advantages and limitations concerning LFIA techniques for SARS-CoV-2 biosensors. Advanced tools for clinical diagnostics discriminating numerous analytes, including viruses and antibodies were explained. Central pillars for building a realistic tackling of current challenges in diagnostic biosensors were analyzed [11].

Jose Alberto Arano-Martinez et al. elucidated a perspective to explore the future of nonlinear optical processes and machine learning methods to improve the performance of non-invasive biosensors. A straightforward demonstration in the effectiveness of multiphonic interactions assisted by machine learning, neural networks and artificial intelligence in nonlinear systems for SARS-CoV-2 sensing is described. The impact of nonlinearities governed with computer tools and soft computing is emphasized in biosensors suitable for the identification of complex low-dimensional agents, viruses, or biological functions in cells [12].

Original research, critical perspectives and panoramic discussions for envisioning new opportunities in the direction of the progress for SARS-CoV-2 biosensors are analyzed in each of the papers published in this Special Issue.
